# Beyond the horizon: Why space biology is the next great innovation opportunity

**DOI:** 10.1113/EP092651

**Published:** 2025-03-03

**Authors:** Carina Kern, Keith Siew

**Affiliations:** ^1^ LinkGevity Babraham Research Campus Cambridge Cambridge UK; ^2^ London Tubular Centre, Department of Renal Medicine University College London London UK

Space exploration has long captured the imagination of humanity, with technological innovation often taking centre stage. From advanced propulsion systems to intricate robotic explorers, our focus has been on the ‘technological how’ of space travel. Yet, as ambitions extend towards Mars and beyond, a more pressing question arises: how do we ensure that our human biology can withstand the challenges of long‐duration missions? Although we have pushed the frontiers of engineering and physics to take us to new worlds, the frailty of the human body could become the ultimate bottleneck. Addressing this issue is not only a necessity for space programmes, it is also a catalyst for innovation that could transform human healthspan and industries on Earth.

In the USA, NASA's Translational Research Institute for Space Health (TRISH), Human Research Program (HRP), Open Science Data Repository (OSDR) and in Europe, the European Space Agency's (ESA) Life Sciences Working Group (LSWG) and Topical Teams (TT) have pioneered efforts to understand how microgravity, cosmic radiation and other space‐specific stressors affect human biology. These programmes have delved into areas such as muscle and bone degeneration, immune suppression and even the impact of isolation on mental health. However, substantial biological challenges persist. With the Terrae Novae Strategy 2030+ and NASA's Artemis missions to the Moon and beyond, sights are now set on the ambitious goal of a crewed mission to Mars, potentially as early as the mid‐ to late 2030s. However, the Martian atmosphere is an exceptionally harsh environment: extremely cold, devoid of oxygen and rich in carbon dioxide, all of which make it seemingly inhospitable to human life. Without targeted funding and strategic initiatives, this vision of long‐duration missions could remain out of reach.

## 1 Space biology: An untapped opportunity

**FIGURE 1:**
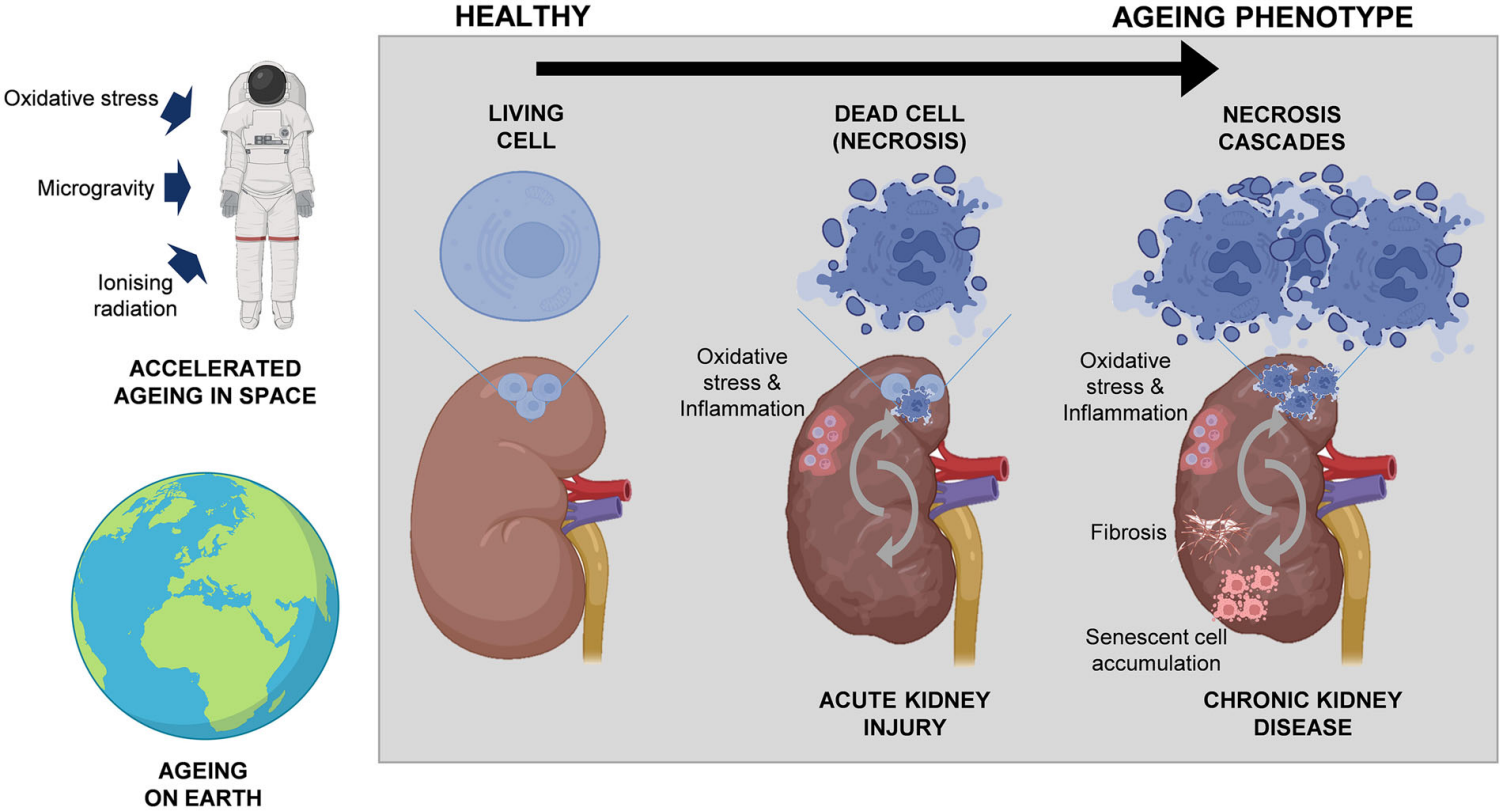
Understanding ageing on Earth through accelerated ageing in space as an untapped opportunity. Ageing in space may mimic ageing on Earth, making mechanistic understanding essential. One of the most susceptible organs to cellular necrosis and ageing on Earth is the kidney, which is linked to acute kidney injury and chronic kidney disease. Kidney deterioration is also likely to be one of the greatest biological challenges for long‐duration space missions (Siew et al., 2024; Ferenbach and Bonventre; 2015).

The potential of space biology extends far beyond the confines of spacecraft. In the UK, initiatives such as the UK Space‐Comm Expo are beginning to draw attention to the intersection of life sciences and the space sector. However, much more can be done to unlock the full potential of this field. The UK government has set enterprising goals for its space sector, but greater emphasis on biological research could both enhance human resilience in space and create new commercial opportunities on Earth. For example, technologies developed to combat kidney dysfunction and muscle and bone atrophy in microgravity could be adapted to improve treatments for kidney disease, sarcopenia and osteoporosis in human ageing (Figure 1).

This intersection of space and biology also offers exciting opportunities for companies willing to think laterally. Many innovations originally developed for space exploration, such as advanced imaging technologies and materials science breakthroughs, from microchips to long‐life tyres, camera phones and cordless headphones, have become transformative for Earth‐based industries. Likewise, the biological interventions required for long‐duration missions could revolutionise sectors such as biomedicine, food preservation and environmental health.

## 2 Encouraging bold steps in space biology

To realise the potential of space biology fully, two key actions are needed.

First, governments (particularly in the UK) must recognise the strategic importance of funding research focused on human biology in space. Although the USA and Europe have made strides with programmes such as TRISH, HRP, OSDR, LSWG ant TTs, the existing UK biomedical infrastructure makes it ripe for dedicated initiatives in this field with equal potential. Increased investment in biological research for space exploration could foster collaborations between academia, industry and space agencies, creating a robust ecosystem of innovation. Moreover, this investment would not only benefit space exploration; it would also drive advancements in healthcare, biotechnology and materials science on Earth.

Second, companies should view space as an untapped market for innovation. By addressing biological challenges, businesses have the opportunity to develop dual‐use technologies with both terrestrial and extraterrestrial applications. The potential rewards, both financial and scientific, are enormous. Imagine creating the solutions that enable the first humans to thrive on Mars while simultaneously advancing regenerative medicine, longevity and precision health technologies on Earth.

## 3 Opening doors to a new frontier: kidney disease and ageing as a case in point

For UK companies and academics, entering the space sector often feels more like a serendipitous accident than a typical career path, at least based on our personal experience. For one of us, Keith Siew, a senior research fellow in academia, a chance email (after a shared bottle of wine and goading from a colleague Ben Walsh) led to a massive collaboration between University College London's London Tubular Centre and over 100 researchers from several space agencies (including NASA, ESA, JAXA, CNSA) and research institutes spannning 5 continents. The work (supported in part by the UK Space Agency, Wellcome Trust and Kidney Research UK) led to one of the most in‐depth studies on how spaceflight radiation and microgravity can induce damage and dysfunction in one of our key organs, the kidneys (Siew et al., 2024).

For the other, Carina Kern, Chief Executive Officer of LinkGevity, a UK‐headquartered European and US biotechnology company, the path to space was equally spontaneous. Working on interventions to target ageing and associated disease had led to development of a potential first‐in‐class intervention for kidney disease and ageing‐related degeneration (supported in part by the European Union's Horizon Europe and the UK Government's National Innovation Agency, Innovate UK). This technology happened to catch the attention of NASA's HRP and TRISH's Space‐Health Program, for which typically only 12 global technologies are chosen per year, revealing an entirely new frontier for innovation: space biology (BioWorld, 2025) and (Business Weekly, 2025). Although many US and EU start‐up companies are already actively exploring the potential of the space sector, the UK is only beginning to tap into this promising field. This example underscores that the space sector is much more than rockets and satellites. It is a burgeoning platform for cross‐sector collaboration and technological breakthroughs. For LinkGevity, it represents a unique opportunity to lead in a nascent industry that can drive benefits for humanity, both on Earth and in space. Many other biotech companies could follow suit.

## 4 Looking ahead

The future of space exploration will depend not only on the robustness of spacecraft but also on the resilience of the human body. As we stand on the brink of interplanetary exploration, addressing the biological challenges of space should be a shared global priority. From mitigating necrosis to ensuring long‐term physiological health, space biology holds the key to unlocking the potential of humanity beyond Earth's atmosphere.

For governments, this is a chance to lead by example, fostering innovation that spans sectors and boundaries. For companies, it is an opportunity to think creatively and boldly, leveraging the challenges of space exploration to drive transformative solutions for life on Earth.

As we push the boundaries of the final frontier, let us remember that the ultimate success of our missions might well depend on the biology we bring with us and the innovations we develop on our way to the stars.

## AUTHOR CONTRIBUTIONS

All authors contributed equally to the manuscript. This editorial did not receive any funding from public, commercial, or not‐for‐profit organizations. Figure 1 was created partially in BioRender. Kern, C. (2025).

## CONFLICT OF INTEREST

C.K. is CEO at LinkGevity; K.S. is NASA SciX Lead Ambassador.

## FUNDING INFORMATION

None.
